# Monitoring how changes in pedagogical practices have improved student interest and performance for an introductory biochemistry course

**DOI:** 10.1002/2211-5463.12409

**Published:** 2018-03-15

**Authors:** Yannis Karamanos, Catherine Couturier, Viviane Boutin, Caroline Mysiorek, Aurélie Matéos, Sylvie Berger

**Affiliations:** ^1^ Faculté des Sciences Université d'Artois Lens France; ^2^ LBHE, Université d'Artois Lens France; ^3^ RECIFES, Université d'Artois Arras France; ^4^ SUPArtois Université d'Artois Arras France; ^5^ Faculté des Sports, Université d'Artois Liévin France

**Keywords:** biochemistry, innovation, learning, pedagogical practices

## Abstract

This study describes feedback on the effects of changes introduced in our teaching practices for an introductory biochemistry course in the Life Sciences curriculum. Students on this course have diverse educational qualifications and are taught in large learning groups, creating challenges for the management of individual learning. We used the constructive alignment principle, refining the learning contract and re‐drafting the teaching program to introduce active learning and an organization of activities that promotes the participation of all the students and helps their understanding. We also created teaching resources available through the university virtual work environment. Our research aimed to measure the effects of those changes on the students’ success. Monitoring of the student performance showed a continuous increase in the percentage of students who passed the course, from 2.13% to 33.5% in 4 years. Analysis of student perceptions highlighted that the teaching methodology was greatly appreciated by the students, whose attendance also improved. The recent introduction of clickers‐questions constituted a complementary leverage. The active involvement of the students and better results for summative assessments are altogether a strong motivation for teaching staff to continue to make improvements.

**Abbreviations**
Bac: baccalauréat
MCQ: multiple choice question
neo‐Bac: Bac obtained the year of first enrolment
PA: participation in activities
SET: student evaluation of teaching

This study refers to the teaching of biochemistry at the beginning of the Life Sciences curriculum at Faculté des Sciences, Université d'Artois. Biochemistry allows students to understand how living organisms function at the molecular level. Two of the biochemistry courses of the curriculum are mandatory, namely, one introductory course during year 1 (L1) of the bachelor degree – ‘license’ in the French context – and a second during the following year (L2). The work reported herein focuses on the introductory course. Why worry about our practices? The figures for previous years, including the academic year 2013–2014, were clear and alarming: very low rates, of the order of a few per cent, of students passed the course, that is, obtained an average mark of ≥ 10/20. Students reported that they ‘feared’ and even ‘hated’ biochemistry, mostly at the beginning of the curriculum. Many students declared that they failed their semester because of biochemistry. Thinking this is not inevitable, we challenged ourselves to improve the students’ motivation and interest in biochemistry and to promote academic success.

It should be noted that there is no selection process for acceptance into a French university: the secondary education diploma, the baccalauréat (Bac; a high school diploma, equivalent to A levels) is sufficient, even if it does not fit with the projected studies. The French high school (lycée) covers the last 3 years of secondary education and prepares pupils for the national Bac examination. There are three types of Bac: (a) a general Bac leading to long studies, namely university, elite schools and business schools, (b) a technological Bac leading to direct entry into the job market or the continuation of specialized technological studies, and (c) a vocational Bac more adapted for young people intending to work in manual or clerical jobs. Thus the diversity of students is high and the management of individuals in large learning groups arises. In addition, the French grading system is quite different from other countries as the low marks obtained at one teaching unit may be compensated by high marks in another unit. Students develop strategies to optimize the work load in favor of a selected number of teaching units and thus they are less interested in others.

After setting up of the ‘Service Universitaire de la pédagogie’ at Université d'Artois, SUPArtois (Teaching and Learning Support Service) during the academic year 2013–2014, and with the help of academic advisors, we initiated student evaluations of teaching, in the sense of an evaluation of the logistical means and resources (technical, human, etc.) for a teaching scenario, not of the teachers themselves. Thus, this step was felt by the teaching staff to be an approach supporting faculty pedagogical development 1, not an assessment of their performance 2.

We used the constructive alignment principle, refining the learning contract and re‐drafting the teaching program to introduce active learning and an organization of activities that promotes the participation of all the students and helps their understanding 3. We defined the learning targets or learning objectives 4, 5 by contextualizing them in the framework of the curriculum and also in ways the students could adopt them as their own 6. We have consecutively modified the teaching activities with the aim of making the students active participants in learning and jointly adapted the learning assessment to allow students to measure their progress all through the semester 7, 8. Our research aims to measure the effects of those changes on the students’ success.

## Material and methods

### Organization of the course

The course ‘General biochemistry: the molecules of life’, organized during the spring semester, is taught to 300–400 students, divided into sections of on average 180 students for classes in lecture halls and into groups of 36 students for tutorial classes. The activities are scheduled for a 12‐week period that includes two 1.5‐h sessions per week in the lecture hall and one 1.5‐h session in tutorial classes. The teaching team has six members, four of them permanent faculty. Profiles of student cohorts are presented in Table 1.

**Table 1:** Characteristics of the student cohorts. Students that filled in the SET form were among those present at the summative assessment. n.d.: not determined

Characteristic	Academic year			
	2013–2014	2014–2015	2015–2016	2016–2017
Number of students in the semester	315	410	343	328
Students with neo‐Bac (%)	56.8	65.6	57.1	57.6
Repeaters (%)	25.4	21.9	32.4	21.4
Absenteeism on week 1 of the course (%)	n.d.	37.8	32.4	26.1
Absenteeism at summative assessment (%)	21.6	19.8	25.6	19.4
Students that filled in the SET form (%)	82.2	88.5	97.9	97.6
Success rate, students present at assessment (%)	2.13	18.4	33.7	33.5
Success rate, engaged students (%)	n.d.	50.0	48.0	56.0

### Pedagogical practices and their changes

Before the academic year 2013–2014 the courses were taught without using technology and without trying to make students active participants. The learning contract was not formalized but the content of the course and the plan were explained at the beginning of each chapter. No formative assessment was used. A few teaching resources, i.e. the figures, were made available through the university virtual work environment using moodle, a learning platform designed to provide educators, administrators and learners with a single robust, secure and integrated system to create personalized learning environments (moodle.org). The grading plan included only summative assessment.

Changes in our practices started in 2014–2015, with a definition of the learning targets for each chapter, a choice of activities that facilitate learning and an explanation of the style of summative assessments. Those modifications brought the members of the teaching team to profoundly analyze the content in order to achieve a coherent assembly of learning targets, content, activities and learning assessment. Thanks to this constructive alignment 3, the introduction of teaching sequences in the lecture hall led to students becoming active participants through activities such as peer instruction sequences 9 – answer a question, then discuss with neighboring students, then with all the class – that foster interactions between students and the teaching team 6, 10. The same year we expanded the online activities through moodle. We progressively posted new teaching resources such as (a) multiple choice questions (MCQs) for formative assessment, (b) summary tables, (c) mini‐videos on particular difficult concepts or aspects of the course (e.g. monosaccharide conversion from the cyclic to the linear form; see Video S1), (d) exercises to prepare tutorials, and (e) a collection of summative assessment questions. A dedicated forum in moodle allowed students to ask questions and to answer the questions of other students in preparing for the tutorials. The key‐words are ‘to be active’ and ‘interactions’. The grading plan included a mark for the active and efficient participation in activities (PA). At the end of this academic year we were able to publish the learning contract as a whole for the first time.

In 2015–2016, the first course was devoted to the analysis of the learning contract (Data S1 in English and Data S2 in French), discussions on the content with peers and with the teacher, and the use of clicker questions. We asked the students to accept the terms of the learning contract, in particular concerning the overall organization and the amount of work input. We tried to improve the understanding of what a student is supposed to do to guarantee success in this course. Additional resources were launched in moodle. Clicker questions were used for formative assessment 11 during courses in the lecture hall, after completion of a particular chapter and at the end of the semester.

In 2016–2017 the activities were carried out as in 2015–2016; in addition (a) ‘chat activity’ (real‐time synchronous discussion) sessions were carried out via moodle allowing students to ask questions in order to revise the course and (b) clicker questions were added in all the courses in the lecture hall and in selected tutorials. Peer‐instruction sequences were frequently used 12. When MCQs are used in the lecture hall or in tutorials, they do not address the level of factual recall (which encourages superficial learning) but rather encourage fruitful exchanges in class (Data S3).

### Research methodology

To study in detail the effects of changes in the pedagogical practices on learning, we implemented two complementary approaches, the monitoring (a) of student performance and (b) of student perceptions.

The monitoring of student performance allows us to measure the changes over academic years of the learning assessment results, assuming that those results represent a reliable indicator of learning performance. From academic year 2014–2015 the grading plan was as follows. There was a mark for the active and efficient PA, relating to both in‐class and online activities, accounting for 10% of the final mark. This incentive assessment is ternary: 0/20 when absent or present at less than half of the activities, 10/20 when present at more than half of the activities and 20/20 when present and active, that is, answering questions, actively participating in tutorials and using the moodle resources (forum, chat, MCQs, etc.). The weak influence of the PA mark on the average is due to the fact that the organization of lectures and tutorials is formative‐assessment oriented, with the aim of helping students to reach the learning targets. We thus assume that when students understand the concepts they will be able to pass the examinations. Two partial examinations, one in mid‐term (EX1) and one at the end (EX2), account for 45% each. EX1 and EX2 are written tests on the course and tutorials, with exercises and questions. Students must be able to demonstrate their knowledge and understanding of the structure of biological macromolecules, the simple links that constitute them and their main physical and chemical properties. The questions are, unless specified, single answer questions and the students are asked to argue their answer.

The monitoring of student perceptions, using a satisfaction survey, allows better understanding of how the students view novel practices. This student evaluation of teaching (SET) is anonymous, and carried out on a voluntary basis, in two steps, the first after 4 weeks of teaching (intermediate SET; Data S4) and the second during the summative assessment. The paper forms are retrieved at the end of the lecture or assessment period. The final SET (Data S5), being more detailed, allows for a thorough assessment. It includes (a) 14 closed questions on the organization of the activities, learning targets, learning assessment, interest in biochemistry, and pedagogical practices, using a Likert‐type scale where the middle ‘neutral’ option is removed 13; (b) two questions on the student's profile; and (c) two open questions.

## Results and Discussion

### Analysis of the student population

**Figure 1:**
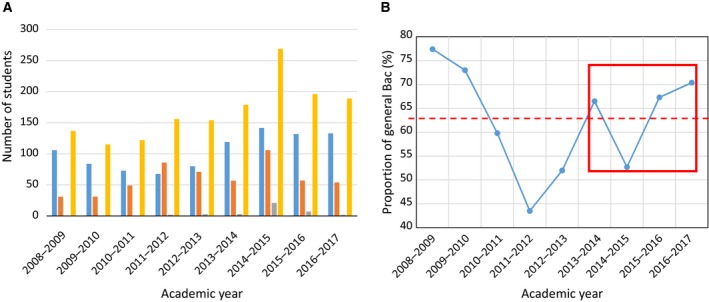
Analysis of the student population. (A) Distribution of the type of neo‐Bac for students enrolled in the first year (L1) of the Life Sciences curriculum. General Bac (blue), technological Bac (orange), vocational bac (gray), total (yellow). (Source: http://www.univ-artois.fr/Formations/Observatoire-de-la-Vie-Etudiante/Reperes-statistiques.) (B) Multi‐year monitoring of the proportion of students (%) with a general Bac among the neo‐Bac. The red frame period represents the period covered in this study. The dashed red line corresponds to the average value of 9 years’ observation (63 ± 11%).

The enrolment varied from year to year (Table 1) depending on the fluxes of students that passed their Bac the year of the first enrolment (‘neo‐Bac’) and the proportion of repeaters. We observed a diversity of types of Bac the students hold and the presence of a significant proportion of technological and more recently a minor proportion of vocational Bacs (Fig. 1A). After a continuous and significant decrease in the proportion of students with a general Bac until 2011–2012, there was relative stability (Fig. 1B). Hence, we can assume that the results of learning assessment and of the SET could not be correlated to the variations of the Bac type.

**Figure 2:**
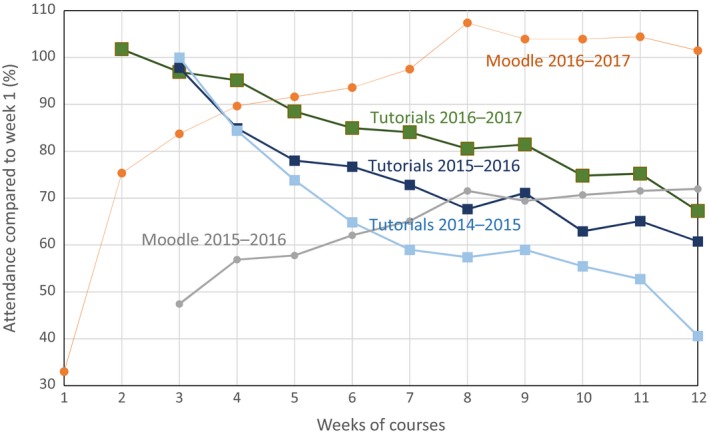
Weekly monitoring of the attendance at tutorials during three academic years and of the voluntary enrolment moodle for 2016–2017. Tutorials started on week 3 in 2014–2015 and 2015–2016 and on week 2 in 2016–2017.

A systemic absenteeism was observed, reaching almost 40%, as early as week 1, for both the activities in the lecture hall and the tutorials. The absenteeism slightly increased during the semester; in parallel we observed a significant increase of participants in the activities available in moodle, with a maximum on the days preceding examinations. Indeed, since the learning targets and the content of the course are clearly described in the learning contract, the students can work outside formal courses to prepare the summative assessment (EX1 and EX2). Moreover, the pedagogical resources in moodle could be used in this regard, and we can see (Fig. 2) that the voluntary enrolment to moodle resources rose from 70% in 2015–2016 to 100% in 2016–2017. Over the academic years, the attendance in tutorials has improved, 10% per year (Fig. 2), but a dropping out was observed in the last 3 weeks, prompting us to review the organization of teaching in this period when all teachers simultaneously ask students for work. This could be addressed by improving the organization of the activities, shared by the teaching staff of all the teaching units. This is more likely to be implemented if a program approach is used.

Absenteeism decreased for summative assessment, and this could be explained by the fact that in the French context, the participation of grant‐holders is mandatory at assessment. Despite this fact we noted that one‐third of the students obtained marks ≤ 4/20. We might be tempted to say that this is a direct consequence of the French regulation (Article 16 de l'arrêté du 1er août 2011, relatif à la licence) for obtaining the ‘license’ degree: the French grading system is quite different from other countries as the low marks obtained at one teaching unit may be compensated by high marks in another unit. Students develop strategies to optimize the work load in favor of a selected number of teaching units and thus they are disinterested in others.

### Student perceptions: student evaluation of teaching

**Figure 3:**
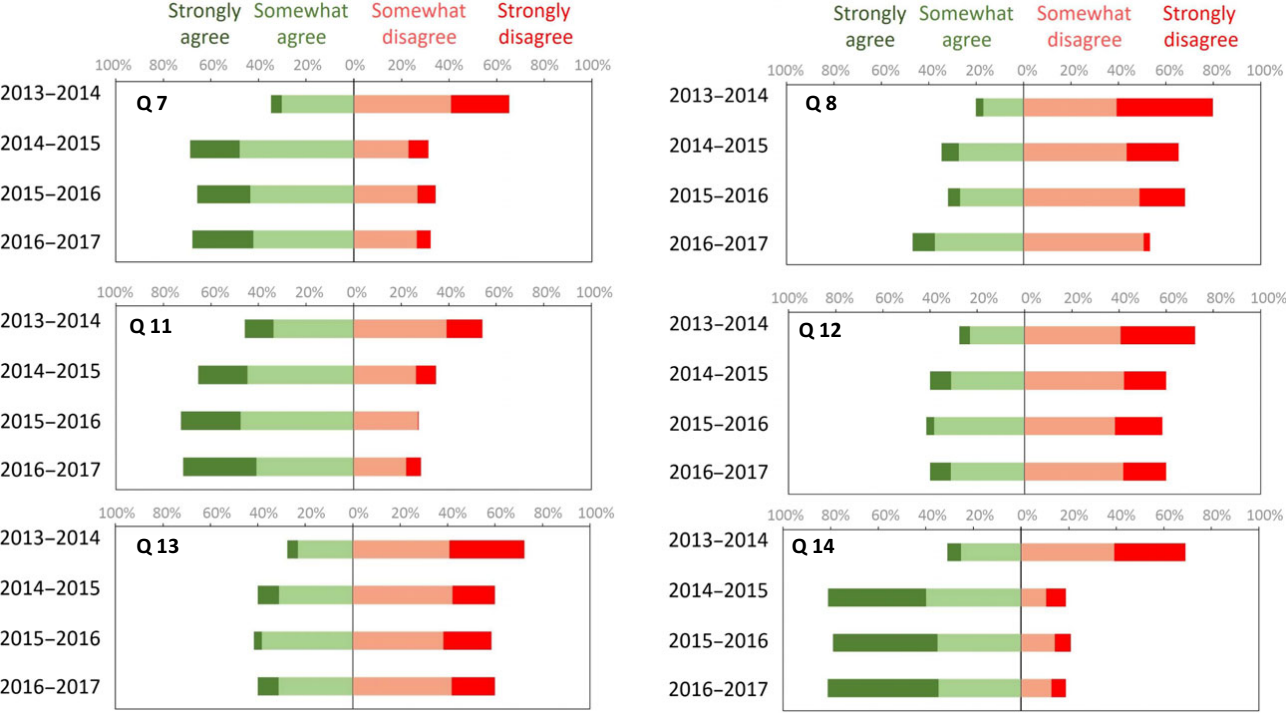
SET during four academic years. Answers to selected questions concerning the sections ‘knowledge/skills taught’ (Q7 and Q8) and ‘interest in teaching/pedagogical methods’ (Q11–Q14). Q7: I believe this course allowed me to progress; Q8: I believe this course prepares me for future professional life; Q11: I understood the significance of this course for the curriculum; Q12: I was led to use the content of this course to resolve practical problems and in interpreting real situations; Q13: This course developed my interest in biochemistry; Q14: The resources in moodle helped me with learning.

During the final SET in 2013–2014, the students expressed positive views for all the questions on the ‘organization of the course’ (questions 1–6, data not shown) and globally negative views for the questions on ‘knowledge/skills taught’ and ‘interest in teaching/pedagogical methods’. For example, they answered 34.7% ‘strongly agree’ + ‘somewhat agree’ for question 7, ‘I believe this course allowed me to progress’; 20.1% for question 8, ‘I believe this course prepares me for future professional life’; and on average 28% for questions on the ‘interest on teaching/pedagogical methods’ (questions 11–14; Fig. 3). Given the comparative results for the following three academic years we can notice (question 11) a clear increase of students’ understanding of the significance of biochemistry in the curriculum. Even the students’ perception of question 8, ‘I believe this course prepares me for future professional life’, which may appear abstract, has gradually evolved. We can observe that the students’ perceptions about question 12, ‘I was led to use the content of this course to resolve practical problems and in interpreting real situations’, and question 13, ‘This course developed my interest in biochemistry’, have not changed over the years. This is of particular interest and a challenge that leads us to rethink the learning targets and to give more sense to the teaching of biochemistry by connecting it to real life.

The SET, done on paper forms each academic year, was filled out by the majority of the students present at summative assessment (Table 1). The anonymous forms were manually analyzed, including open questions, since not all of the students expressed views. To the question ‘What are the points of lectures/tutorials/moodle resources that you are most interested in?’, the students replied, to appreciate the real‐life aspects of the course, the mini‐videos in moodle focused on difficult aspects of the course, and the time the teacher spends in answering all the questions raised. In 2015–2016 the students greatly appreciated the introduction and development of formative assessment using clicker questions. The purpose of incorporating clicker questions was to increase student engagement in the hope of consequently increasing their performance 14. During this academic year the change was already felt during the intermediate SET (week 4), in particular for the peer instruction sequences. The students underlined the effectiveness of this approach and the use of the resources in moodle, both of them having certainly contributed to the engagement of many more students. Examples of sentences frequently cited are: ‘I enjoyed the interactions with other students’, ‘please use more questions with clickers in the course’, and ‘the mini‐videos in moodle help in understanding the corresponding concept or a particular aspect of the course’.

### Student performance (success rates)

The course is declared passed when an average mark of ≥ 10/20 is obtained for the grading plan. The success rate is the percentage of students who passed the course. What should be the reference to calculate the success rate? The number of enrolled students, the number of students present on examinations or the number of students really engaged in a learning process? In this study, due to the absenteeism, we decided to use the success rate based on the number of students present during summative assessment (EX1 and EX2) instead of the number of enrolled students (Table 1). In addition starting from academic year 2014–2015 we have recalculated the success rate for students that are engaged in a learning process (the number of engaged students is deduced by the PA component of the grading plan, namely students with a PA mark of 10/20 and 20/20).

**Figure 4:**
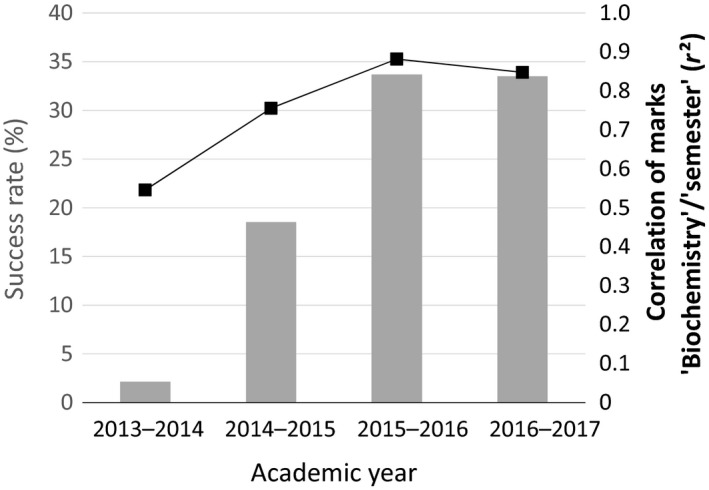
Change of the success rate (%) for the biochemistry introductory course during the first year of license (gray columns) and correlation (*r*²) between marks obtained by the students for biochemistry and for the corresponding semester (black squares).

We can correlate the students’ perception of the significance of the course for the curriculum (Fig. 3, question 11) to the changes of success rates, which significantly increased, from 2.13% to 33.5% in 4 years of observation (Fig. 4). An improvement in the correlation of the results for this course with the results of the corresponding semester was observed; the coefficient of determination (*r*²) progressively reached 0.9.

We realize that the success rate is not a direct and fair reflection of the student population. It represents 40% for students with a general Bac versus 4% for students with a technological or vocational Bac. As noted previously, many students do not engage in a learning process. Taking into account this observation, the recalculated success rate for student engaged in a learning process is higher, 56% in 2016–2017 (Table 1). When we add the number of students that obtained a mark between 8 and 10/20 (which could be considered equivalent to the ECTS FX grade of Fail – some more work is required before the credit can be awarded) the success rate reaches 74%.

Finally, what we observed with our students agrees with the statement, ‘Today, in higher education, it is possible to talk about innovation both in terms of product (student success and reputation of the institution) and in terms of process (commitment and perseverance of students). These two approaches are no doubt complementary, yet they implicitly carry with them the notion of progress’ (translated from 15).

### Conclusions and future prospects

We wanted to change our pedagogical practices in order to improve the students’ interest in biochemistry and promote their success, which was very low for the academic years prior to 2014–2015. After redefining the learning targets, we gradually adapted the learning assessment, revisited the contents of the course and introduced interaction between students and between students and teachers, as well as interactivity using Information and Communication Technologies (ICT). We also developed teaching resources available in the moodle platform through the university virtual work environment. Our approach has been inspired and is in line with the six levers to enhance the learning of higher education students reported by Marianne Poumay 16, namely (a) to improve the pedagogical alignment, (b) to make the student an active learner, (c) to enhance the value of the activities, (d) to increase the feeling of mastery, (e) to give the student more control, and (f) to introduce the use of ICT. It was recently proposed to consider innovation as ‘a process, characterized by generation, acceptance and implementation of change through new ideas, processes, products or services, and conducted by actors in a collective development’ 15. The innovative aspect of our approach is on the one hand the strong involvement of the teaching team and on the other hand the empirical, longitudinal and thorough verification of the effects of the changes of the pedagogical approach. Our study shows a clear increase in the interest of students in biochemistry as well as the relevance of this course for their curriculum; however, we believe that we have reached a maximum with the means that we are currently implementing. The student success rate has been significantly improved from 2.13% to 33.5% among those present at assessment, but we have probably reached a saturation point, in the sense that we cannot influence more students that are not engaged in a learning process if we do not improve other organizational and pedagogical aspects.

We will continue to change our practices for teaching biochemistry for all the levels of the curriculum as well as the development of teaching resources adapted to interaction/interactivity. We have seen a marked improvement in the active participation of students in lecture classes. Unfortunately, we have also noticed that not all of the students prepare courses before class. On the basis of the first experience of using clicker question sequences for courses in the lecture hall and after the first attempt with one tutorial in 2016–2017, we decided to extend their use during other tutorials and introduce team work to implement the method ‘tutorials without corrections’ based on a structured progression of exercises where the instructor never give a correction element 17. Thus, the student has no choice but to cooperate with peers to find the answers, which are finally validated by the instructor who stands back, of course, but plays an essential role of session manager and learning facilitator.

We agree that what teachers do has a great impact on learning, but this is not the end of the story, as what students do to learn is critically important 18. To further improve the engagement of students and facilitate learning in biochemistry, it seems that we should raise awareness of the interconnections of biochemistry with other disciplines, and instill into students that biochemistry will allow them to understand biological processes. Moreover we should give more sense to the teaching of biochemistry by connecting it to real life as expressed by Viau's motivation theory 19. We also believe that there should be a better accounting of the threshold concepts, that is concepts that, when mastered, represent a transformed and integrative understanding of a discipline without which the learner cannot progress 20.

Our future prospects for research are (a) to continue our study of measuring the effects of the new teaching methods implemented, (b) to try to understand what is being played out from the point of view of the teachers who transform their practices, (c) to better understand the perception of the students by means of individual and collective interviews, and (d) to follow the changes of the indicators on a cohort of students throughout the 3 years of the license curriculum.

## Author contributions

YK conceived the project and wrote the draft manuscript; YK, CC and VB designed the project; CC and VB discussed all the pedagogical issues; VB observed several courses given by YK in the lecture hall and reported; YK and SB teach in the lecture hall; YK, CM and AM teach in tutorials. YK, SB, CM and AM acquired the data; all the authors analyzed and interpreted the data. All the authors read and amended the manuscript to obtain its final form.

## Supporting information


**Video S1.** Monosaccharide: conversion from the cyclic to the linear form.

**Data S1.** Learning contract of the course, in English.

**Data S2.** Learning contract of the course, in French.

**Data S3.** Examples of MCQs, in English.

**Data S4.** Form of the intermediate SET, in English and French.

**Data S5.** Form of the final SET, in English and French.
