# Supplementary material for: Monitoring how changes in pedagogical practices have improved student interest and performance for an introductory biochemistry course
Source: FEBS Open Bio. 2018 Mar 15;8(4):494–501. doi: 10.1002/2211-5463.12409 (PMC5881536; doi:10.1002/2211-5463.12409)
Supplement: Supplementary file 2 — Data S1. Learning contract of the course, in English. [file FEB4-8-494-s002.pdf]

Université d'Artois - Faculté des Sciences

Licence Sciences de la Vie

Teaching Unit (TU)

General Biochemistry: The molecules of life (BBM1)

Responsible: Yannis Karamanos

Members of the teaching team:
Lectures : Yannis Karamanos, Sylvie Berger
Tutorials : Aurélie Matéos, Barbara Déracinois, Laurent Finet
Caroline Mysiorek, Yannis Karamanos

Learning contract

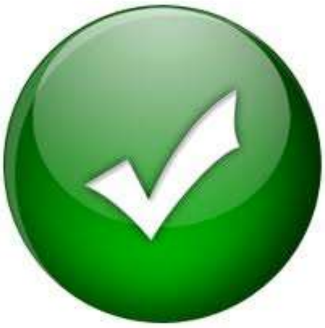

Table of contents

Foreword 2

Prerequisites 2

Organisation 2

Learning targets and content elements 3

I. General introduction : 3

II. Proteins-Enzymes : 3

III. Lipids : 5

IV. Carbohydrates : 5

V. Nucleic acids : 6

Assessment methods of learning 8

Recommended readings 8

Foreword

Biochemistry can be defined as the science of the chemical bases of life. Since the cell is the structural unit of living systems, biochemistry can also be defined as the science that studies the chemical constituents of living cells and the reactions and transformations they undergo. The TU BBM1 is designed to enable students to discover the composition of the macromolecules constituting the cells, their structures and the structure of the simpler links that constitute them and their main physical and chemical properties.

Prerequisites

The knowledge acquired in high school via the general bac

Organisation

This teaching requires about 120 hours of work, including attendance at classes and tutorials and especially your personal work.

The learning will be done through:

- Lectures: 22 sessions from 1h to 1h15 (2 sessions per week from week 1): 1 introduction, 7 on proteins and enzymes, 3 on lipids, 8 on carbohydrates and 3 on nucleic acids
- Tutorials: 1 session of 1h and 10 sessions of 1h30 (1 session per week starting from week 2): 3 on proteins, 1 on enzymes, 1½ on lipids, 4 on carbohydrates and 1½ on nucleic acids
- Personal work (study in textbooks, resolution of exercises before tutorials, active participation in questions during class and tutorials, etc.)
- Discussions with other students
- The use of moodle BBM1's educational resources (answers to tutorials, revisions...)

Rules during sessions:

- When you enter the classroom, you agree to respect your colleagues and teachers, to be courteous, not to eat during the class, to be silent during presentations, not to talk to each other for no reason.
- You will be asked to participate in the various activities proposed by the teachers, for example reading documents before the class, answering questions before or during the class, discussing with your close neighbours to refine your arguments etc.
- You can ask questions related to the course! You can either raise your hand to ask your question (strongly desired) or write it on a small paper and drop it at the end of the course or send it by email to bioch@univ-artois.fr. In the last two cases the answers will be given at the beginning of the next class. They will all be listed, with the answers, on moodle's BBM1 space.

Learning targets and content elements

The learning targets and content elements are presented by chapter:

I. General introduction :

At the end of this course you should be able to:

(a) Explain the purpose of biochemistry

(b) Measure how sequencing of the human genome has given rise to many disciplines

(c) Describe the properties of water and its importance for the structuring of biological macromolecules

(d) Explain the relationship of pH with acidity and alkalinity and what characterizes weak acids and bases

Course contents:

Definition
Objectives of biochemistry
Impact of sequencing the human genome
Relationship between biochemistry and medicine
Water structure Importance of water
Recall of the concept of pH
Dissociation of weak acids and bases

II. Proteins-Enzymes :

At the end of this course you should be able to:

(a) Explain the presence of 20 amino acids as structural units of peptides and proteins.

(b) Define the structure of an α-amino acid and its different forms in solution in water (the structure of the 20 amino acids is required).

(c) Explain how amino acids are bound to form peptide bonds

(d) Explain why peptide bonds are rigid and flat.

(e) Argue the fact that proteins have unique sequences that are specified by genes

(f) List the ionisable groups of amino acids

(g) Explain how pH, pK and pHi can be used to predict the mobility of an amino acid in a continuous electric field

(h) Describe the contribution of R-groups of amino acids in the structure of peptides and proteins

(i) Describe how the amino acid sequence specifies the 3-D structure of proteins that specifies their biological function

(j) Identify the main known types of secondary structures and explain more elaborate structures

(k) Describe the nature of the forces that stabilize the protein structure in space
(l) Identify and describe the interactions of CO, CO_2 and H_2O with haemoglobin
(m) To know the notion of chemical reaction and the interest of catalysis
(n) To understand the notion of molecular catalyst
(o) Explain the specificity of enzyme reaction and substrate
(p) Explain why laboratory measurements of the speed of a reaction are made under initial velocity conditions
(q) Understand that substrate concentration affects reaction rate and explain the mathematical modelling of this relationship between reaction rate and substrate concentration

Course contents:

A) Introduction
   1. Definition
   2. Importance and role of proteins
   3. General formula - classification

B) Amino acids
   I . Definition
   II. Classification – presentation
   III. Physical properties
      1. Aspect
      2. Solubility
      3. Stereoisomers
      4. Absorption in the ultraviolet
      5. Ionisation
   IV. Main reactions

C) Peptide bond

D) Peptides

E) Proteins

Content of the tutorials:

Tutorial Proteins 1: deals with the use of molecular models to familiarize with Fischer projection, with the formulas of some amino acids

Tutorial Proteins 2: deals with peptide diversity, the general formula of amino acids, the calculation of their molar mass, the notion of absorbance and the Beer-Lambert law

Tutorial Proteins 3: focuses on the ionization of amino acids and the contribution of side chains to protein ionization, the relationship between the overall charge of a protein and their isoelectric point, and the interactions of haemoglobin with O_2. , CO and CO_2.

Tutorial Enzymes: focuses on enzymes, the interest of working in initial velocity, an example of enzymatic kinetics as well as the graphical exploitation of the Michaelis-Menten equation.

III. Lipids :

At the end of this course you should be able to:

(a) Define simple or complex lipids and identify the lipid classes of each group

(b) Specify the structure of saturated and unsaturated fatty acids and explain how chain length and degree of unsaturation affect melting temperature

(c) Describe the general structure of acylglycerols and especially triacylglycerols and their main properties

(d) Describe the general structure of phospholipids and sphingolipids

(e) Understanding the role of lipids in biological membranes

(f) Understanding the organization of biological membranes

(g) Identify the four main classes of lipoproteins and the types of lipids they carry

(h) Illustrate the structure of a lipoprotein particle and describe the characteristic apolipoproteins for each class

Course contents:

A) Introduction
B) Lipids as energy reservoirs
   Fatty acids
   Acylglycerols
   Waxes
C) Lipids of biological membranes
   Phospholipids
   Sphingolipids
   Cholesterol
D) Biological membranes
   Properties of membrane lipids
   Membrane proteins
E) Lipoproteins : transport and storage of lipids

Content of the tutorials:

Tutorial Lipids 1-2: (one-and-a-half session) focuses on the structures and properties of fatty acids and glycerides as well as the structures of the main membrane lipids and lipoproteins

IV. Carbohydrates :

At the end of this course you should be able to:

(a) Know the structure of the most common carbohydrates and their essential properties

(b) Realize that this class of natural substances is extremely diverse

(c) Explain the meaning of the terms: monosaccharide, disaccharide, oligosaccharide and polysaccharide

(d) Explain the different ways of representing the structures of glucose and other monosaccharides and describe the various types of isomers, pyranose- and furanose-type cyclic structures

(e) Describe the formation of glycosides and the structures of important disaccharides and polysaccharides

Course contents:

A) Introduction
B) Monosaccharides
   Neutral sugars
   Amino sugars
   Acidic sugars (Uronic acids, sialic acids)
   Reactivity of monosaccharides
C) Glycosides
   Glycosidic bond
   Reducing and non-reducing glycosides
   Examples
   Disaccharides
   Oligosaccharides, glycoproteins et glycolipids
   Polysaccharides
   Glycosaminoglycans, proteoglycanes and peptidoglycans

Content of the tutorials:

Tutorial Carbohydrates 1: (half-session) focuses on the use of molecular models to review the Fischer projection, illustrate and understand the structure of linear forms of monosaccharides and their stereochemical relationships.

Tutorial Carbohydrates 2: deals with the characteristics of the monosaccharides, the different isomers and the different representations and projections

Tutorial Carbohydrates 3: focuses on the use of molecular models to understand the spatial structure of monosaccharides, their cyclic forms, determine their most stable conformation

Tutorial Carbohydrates 4-5: (one-and-a-half session) makes it possible to become familiar with the cyclization of monosaccharides and their ring opening, their chemical properties and the structure of glycosides

V. Nucleic acids :

At the end of this course you should be able to:

(a) To be able to write formulas representing the tautomeric forms of a purine and a pyrimidine and to specify the predominant tautomeric form under physiological conditions

(b) Reproduce the formulas of the main nucleotides present in DNA and RNA and those of the less frequent nucleotides of 5-methylcytosine, 5-hydroxymethylcytosine and pseudouridine (Ψ)

(c) Represent the binding of D-ribose and 2-deoxy-D-ribose to a purine or pyrimidine, to name the type of bond between the ose and the base.

(d) Number the C and N of a pyrimidine or purine nucleotide without forgetting the symbols n 'of the atoms of the dare

(e) Knowing the difference in energy potential between the phosphoester and phosphoanhydride bonds of a nucleoside triphosphate

(f) Realizing that the polynucleotides are oriented macromolecules composed of mononucleotides connected by phosphodiester bonds 3'→5 '

(g) Understand that in abbreviated notation polynucleotides, for structures like pTpGpT or TGCATCA, the 5 'end is always shown on the left and all phosphodiester bonds are of type 3'→5'

(h) Explain how the secondary structure of DNA (double-stranded helix) is obtained and stabilized

(i) Know the main physical and chemical properties of nucleotides and nucleic acids

(j) Know the different forms of RNA and their role

Course contents:

A) Nucleotides
    - Introduction
    - Structure and properties
    - Pentoses
    - Bases
  - Nucleosides
  - Nucleotides
  - Roles of nucleotides
    - Energy
    - Constituents of co-enzymes
    - Second messengers

B) Nucleic acids
    - Dimensions
    - Primary structure
    - Secondary structure
    - Properties of DNA
    - Different forms of RNA

Content of the tutorials:

Tutorial Nucleic acids 1-2: (one-and-a-half session) relates to the main bases, their keto and enol forms, the constitution of the nucleosides and nucleotides, the molar composition of the DNA in bases, the relation between number of base pairs and molecular mass, the action of some restriction enzymes, the main properties of DNA

Assessment methods of learning

Targets:

Students must be able to demonstrate their knowledge and understanding of the structure of biological macromolecules, the simple links that constitute them and their main physical and chemical properties.

Style:

- The diligent and efficient participation in the activities proposed during the classes, tutorials and moodle's BBM1 space is taken into account for 10% of the mark (three possible marks: absent = 0, assiduous = 10, active = 20).
- Two 1.5-hour written tests on the course and tutorials (one at mid-term and one at the end counting 45% each)
  - Partial exam 1 on parts II. Proteins-Enzymes and III. Lipids (at the beginning of week 8)
  - Partial exam 2 on parts IV. Carbohydrates and V. Nucleic Acids (at the end of the semester)

Note: in session 2 a final exam of 2h, covering all the courses and tutorials, replaces the two marks of the partial exams (so it counts for 90%)

The questions are, unless specified, single answer questions. In the majority of cases you will be asked to argue your answer.

Glossary: Define (just a formal sentence), quote (concise answer with little or no evidence / arguments), describe (cite important points with sentences and with schemas if appropriate), explain (provide reasoning and / or references to theories depending on the context), or summarize (just the essential)

Recommended readings

📖 'Biochimie Générale' de Jacques-Henry Weil, Dunod éditeur

📖 'Biochimie' de Michel Guilloton et Bernadette Quintard, Paul-François Gallet, Dunod éditeur
