# Supplementary material for: Monitoring how changes in pedagogical practices have improved student interest and performance for an introductory biochemistry course
Source: FEBS Open Bio. 2018 Mar 15;8(4):494–501. doi: 10.1002/2211-5463.12409 (PMC5881536; doi:10.1002/2211-5463.12409)
Supplement: Supplementary file 3 — Data S2. Learning contract of the course, in French. [file FEB4-8-494-s003.pdf]

Université d'Artois - Faculté des Sciences

Licence Sciences de la Vie

Unité d'enseignement

Biochimie générale : les molécules du vivant (BBM1)

Responsable : Yannis Karamanos

Membres de l'équipe pédagogique :
Cours : Yannis Karamanos, Sylvie Berger
Travaux dirigés : Aurélie Matéos, Barbara Déracinois, Laurent Finet
Caroline Mysiorek, Yannis Karamanos

Contrat pédagogique

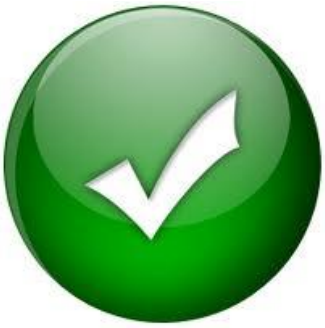

Sommaire

Avant-propos 2

Prérequis 2

Modalités de fonctionnement 2

Apprentissages visés et éléments de contenus des enseignements 3

I. Introduction générale : 3

II. Protéines-Enzymes : 3

III. Lipides : 5

IV. Glucides : 6

V. Acides nucléiques : 7

Modalités d'évaluation des apprentissages 8

Lectures conseillées 8

Avant-propos

La biochimie peut être définie comme la science des bases chimiques de la vie. La cellule étant l'unité structurale des systèmes vivants, on peut aussi définir la biochimie comme la science qui étudie les constituants chimiques des cellules vivantes ainsi que les réactions et transformations qu'ils subissent. L'UE BBM1 est conçue pour permettre aux étudiants de découvrir la composition des macromolécules constituant les cellules, leurs structures et la structure des maillons plus simples qui les constituent ainsi et leurs propriétés physiques et chimiques principales.

Prérequis

Les connaissances acquises au lycée via le bac scientifique

Modalités de fonctionnement

Cet enseignement nécessite environ 120 h de travail, incluant la présence aux cours et travaux dirigés et surtout votre travail personnel.

Les apprentissages se feront à travers :

- Des cours, 22 séances de 1h à 1h15 (2 séances par semaine dès la semaine 1) : 1 introduction, 7 sur les protéines et enzymes, 3 sur les lipides, 8 sur les glucides et 3 sur les acides nucléiques
- Des travaux dirigés, 1 séance de 1h et 10 séances de 1h30 (1 séance par semaine à partir de la semaine 2) : 3 sur les protéines, 1 sur les enzymes, 1½ sur les lipides, 4 sur les glucides et 1½ sur les acides nucléiques
- Du travail personnel (étude dans des manuels, résolution des exercices avant les travaux dirigés, participation active lors des questions pendant les cours et les travaux dirigés etc.)
- Des discussions avec les autres étudiants
- L'utilisation des ressources pédagogiques de l'espace BBM1 de moodle (réponses à des questions de travaux dirigés, révisions..)

Règles lors des enseignements :

- En entrant dans la salle de cours vous vous engagez à respecter vos collègues et les enseignants, à être courtois, à ne pas manger pendant le cours, à rester silencieux lors des présentations, à ne pas parler entre vous sans raison.
- Vous serez sollicités à participer aux différentes activités proposées par les enseignants, par exemple lire des documents avant le cours, répondre à des questions avant ou pendant le cours, discuter avec vos proches voisins pour affiner vos arguments etc.
- Vous pouvez poser des questions en lien avec le cours ! Vous pouvez soit lever la main et poser votre question (vivement souhaité) soit l'écrire sur un petit papier et la déposer en fin de cours soit l'envoyer par messagerie électronique à bioch@univ-artois.fr. Dans

les deux derniers cas les réponses seront données au début du cours suivant. Elles seront toutes répertoriées, avec les réponses, sur l'espace BBM1 de moodle.

Apprentissages visés et éléments de contenus des enseignements

Les acquis d'apprentissage visés et éléments de contenus sont présentés par chapitre :

I. Introduction générale :

A l'issue de ce cours vous devriez être capables de :

(a) Expliquer l'objet de la biochimie

(b) Mesurer comment le séquençage du génome humain a donné naissance à de nombreuses disciplines

(c) Décrire les propriétés de l'eau et de son importance pour la structuration des macromolécules biologiques

(d) Expliquer la relation du pH avec l'acidité et l'alcalinité ainsi que ce qui caractérise les acides et bases faibles

Contenus du Cours :

Définitions
Objectifs de la biochimie
Impact du séquençage du génome humain
Relations entre biochimie et médecine
Structure de l'eau
Importance de l'eau
Rappel de la notion de pH
Dissociation des acides et bases faibles

II. Protéines-Enzymes :

A l'issue de ce cours vous devriez être capables de :

(a) Expliquer la présence de 20 acides aminés en tant qu'unités structurales des peptides et des protéines.

(b) Définir la structure d'un α-acide aminé et ses différentes formes en solution dans de l'eau (la structure des 20 acides aminés est requise).

(c) Expliquer comment les acides aminés sont liés pour former des liaisons peptidiques

(d) Expliquer pourquoi les liaisons peptidiques sont rigides et planes.

(e) Argumenter le fait que les protéines ont des séquences uniques qui sont spécifiées par des gènes

(f) Donner la liste des groupements ionisables des acides aminés

(g) Expliquer comment on peut utiliser la pH, le p*K* et le pHi pour prédire la mobilité d'un acide aminé dans un champ électrique continu

(h) Décrire la contribution des groupes R des acides aminés dans la structure des peptides et protéines

(i) Décrire comment la séquence en acides aminés spécifie la structure 3-D des protéines qui spécifie leur fonction biologique

(j) Identifier les principaux types connus de structures secondaires et d'expliquer des structures plus élaborées

(k) Décrire la nature des forces qui stabilisent la structure des protéines dans l'espace

(l) Identifier et décrire les interactions de CO, CO_2 et H_2O avec l'hémoglobine

(m) Connaître la notion de réaction chimique et l'intérêt de la catalyse

(n) Appréhender la notion de catalyseur moléculaire

(o) Expliquer la spécificité de réaction et de substrat des enzymes

(p) Expliquer pourquoi les mesures en laboratoire de la vitesse d'une réaction se font dans les conditions de vitesse initiale

(q) Comprendre que la concentration du substrat affecte la vitesse de réaction et expliquer la modélisation mathématique de cette relation entre la vitesse de réaction et la concentration de substrat

Contenus du cours :

- A) Introduction
  1. Définition
  2. Importance et rôle des protides
  3. Formule générale - classification
- B) Les acides aminés (ou aminoacides)
  - I . Définition
  - II. Classification – présentation
  - III. Propriétés physiques
    1. Aspect
    2. Solubilité
    3. Stéréoisomerie
    4. Absorption dans l'ultraviolet
    5. Ionisation
  - IV. Principales réactions
- C) Les protides conjugués
- D) Les peptides
- E) Les protéines

Contenus des Travaux dirigés :

LeTD 1 porte sur l'utilisation de modèles moléculaires pour se familiariser avec la projection de Fischer, avec les formules de quelques acides aminés

LeTD 2 porte sur la diversité des peptides, la formule générale des acides aminés, le calcul de leur masse molaire, la notion d'absorbance et la loi de Beer-Lambert

Le TD 3 porte sur l'ionisation des acides aminés et la contribution des chaînes latérales sur l'ionisation des protéines, la relation entre la charge globale d'une protéine et leur point isoélectrique et sur les interactions de l'hémoglobine avec l'O_2, le CO et le CO_2.

Le TD Enzymes porte sur les enzymes, l'intérêt de travailler en vitesse initiale, un exemple de cinétique enzymatique ainsi que l'exploitation graphique de l'équation de Michaelis-Menten

III. Lipides :

A l'issue de ce cours vous devriez être capables de :

(a) Définir ce que sont les lipides simples ou complexes et d'identifier les classes de lipides de chaque groupe

(b) Préciser la structure des acides gras saturés et insaturés et d'expliquer comment la longueur de chaîne et le degré d'insaturation influencent la température de fusion

(c) De décrire la structure générale des acylglycérols et en particulier des triacylglycérols et leurs principales propriétés

(d) Décrire la structure générale des phospholipides et des sphingolipides

(e) Comprendre le rôle des lipides dans les membranes biologiques

(f) Comprendre l'organisation des membranes biologiques

(g) Identifier les quatre classes principales de lipoprotéines et les type de lipides qu'elles transportent

(h) Illustrer la structure d'une particule de lipoprotéine et décrire les apolipoprotéines caractéristiques pour chaque classe

Contenus du cours :

A) Introduction
B) Lipides réservoirs d'énergie
   Les acides gras
   Les acylglycérols
   Les cires
C) Lipides des membranes biologiques
   Les phospholipides
   Les sphingolipides
   Le cholestérol
D) Les membranes biologiques
   Propriétés des lipides membranaires
   Protéines membranaires
E) Lipoprotéines : transport et stockage des lipides

Contenus des Travaux dirigés :

Le TD1-2 (qui se fait sur une séance et demie) porte sur les structures et propriétés des acides gras et des glycérides ainsi que sur les structures des principaux lipides membranaires et les lipoprotéines

IV. Glucides :

A l'issue de ce cours vous devriez être capables de :

(a) Connaître la structure des glucides les plus courants et leurs propriétés essentielles

(b) Réaliser que cette classe de substances naturelles est extrêmement diversifiée

(c) Expliquer la signification des termes : monosaccharides, disaccharide, oligosaccharide et polysaccharide

(d) Expliquer les différentes façons de représenter les structures du glucose et des autres monosaccharides et de décrire les divers types d'isomérie, les structures cycliques de type pyranose et furanose

(e) Décrire la formation des glycosides et les structures des disaccharides et polysaccharides importants

Contenus du cours :

A) Introduction
B) Les monosaccharides
   Oses neutres
   Oses basiques (Osamines)
   Oses acides (Acides uroniques, acides sialiques)
   Réactivité des oses
C) Les osides
   Liaison osidique
   Osides réducteurs et non réducteurs
   Exemples
   Les disaccharides
   Oligosaccharides, glycoprotéines et glycolipides
   Polysaccharides
   Glycosaminoglycanes, protéoglycanes et peptidoglycanes

Contenus des Travaux dirigés :

Le TD1 (qui se fait sur une demi-séance) porte sur l'utilisation de modèles moléculaires pour revoir la projection de Fischer, illustrer et comprendre la structure des formes linéaires des oses et leur filiation.

Le TD 2 porte sur les caractéristiques des oses, les différents isomères et les différentes représentations et projections

Le TD 3 porte sur l'utilisation de modèles moléculaires pour comprendre la structure des oses dans l'espace, leurs formes cycliques, déterminer leur conformation la plus stable

Le TD 4-5 (qui se fait sur 1 séance et demie) permet de se familiariser avec la cyclisation des oses et leur décyclisation, les propriétés chimiques des oses et la structure des osides

V. Acides nucléiques :

A l'issue de ce cours vous devriez être capables de :

(a) Savoir écrire des formules représentant les formes tautomères d'une purine et d'une pyrimidine et préciser la forme tautomère prédominante dans les conditions physiologiques

(b) Reproduire les formules des principaux nucléotides présents dans l'ADN et l'ARN et celles des nucléotides moins fréquents que sont la 5-méthylcytosine, la 5-hydroxyméthylcytosine et la pseudouridine (Ψ)

(c) Représenter la liaison du D-ribose et du 2-désoxy-D-ribose à une purine ou une pyrimidine, de nommer le type de liaison entre l'ose et la base.

(d) Numéroter les C et N d'un nucléotide pyrimidique ou purique sans oublier les symboles n' des atomes de l'ose

(e) Connaître la différence de potentiel énergétique entre les liaisons phosphoester et phosphoanhydride d'un nucléoside triphosphate

(f) Réaliser que les polynucléotides sont des macromolécules orientées composés de mononucléotides reliés par des liaisons phosphodiester 3'→5'

(g) Comprendre qu'en notation abrégée des polynucléotides, pour des structures comme pTpGpT ou TGCATCA, l'extrémité 5' est toujours représentée à gauche et que toutes les liaisons phosphodiester sont de type 3'→5'

(h) Expliquer comment la structure secondaire de l'ADN (hélice double brin) est obtenue et stabilisée

(i) Connaître les principales propriétés physiques et chimiques des nucléotides et des acides nucléiques

(j) Connaître les différentes formes d'ARN ainsi que leur rôle

Contenus du cours :

- A) Les nucléotides
  - Introduction
  - Structure et propriétés
  - Pentoses
  - Bases azotées
  - Nucléosides
  - Nucléotides
  - Rôle des nucléotides
    - Energétique
    - Constituants des co-enzymes
    - Seconds messagers
- B) Les acides nucléiques

Taille
Structure primaire
Structure secondaire
Propriétés de l'ADN
Différentes formes d'ARN

Contenus des Travaux dirigés :

Le TD 1-2 (qui se fait sur une séance et demie) porte sur les principales bases azotées, leurs formes céto et énol, la constitution des nucléosides et nucléotides, la composition molaire de l'ADN en bases, la relation entre nombre de paires de bases et masse moléculaire, l'action de quelques enzymes de restriction, les principales propriétés de l'ADN

Modalités d'évaluation des apprentissages

Objectifs : Les étudiants doivent être capables de démontrer leurs connaissances et compréhension de la structure des macromolécules biologiques, des maillons simples qui les constituent ainsi que de leurs propriétés physiques et chimiques principales.

Forme :

- La participation assidue et efficace aux activités proposées lors des cours, travaux dirigés et sur l'espace BBM1 de moodle est prise en compte pour 10% de la note (trois notes possibles : absent = 0 ; assidu = 10 ; actif = 20).
- Deux épreuves écrites de 1h30 portant sur le cours et les TD (une à mi-parcours et une à la fin comptant 45% chacune)
    - 1ère portant sur les parties Protéines, Enzymes et Lipides (à priori semaine 8)
    - 2nde à la fin portant sur les parties Glucides et Acides Nucléiques

  Remarque : lors de la session 2 une épreuve finale de 2h, portant sur la totalité des cours et TD, remplace les deux notes des épreuves partielles (elle compte donc pour 90%)

Les questions à choix sont, sauf indication contraire, à réponse unique. Dans la majorité des cas il vous sera demandé de justifier votre réponse.

Glossaire : Définir (juste une phrase formelle), citer (réponse concise avec peu ou pas de preuves/arguments), décrire (cela demande de citer les points importants avec des phrases et avec des schémas si approprié), expliquer (apporter un raisonnement et/ou des références à des théories en fonction du contexte), ou résumer (juste l'essentiel)

Lectures conseillées

🕮 'Biochimie Générale' de Jacques-Henry Weil, Dunod éditeur

🕮 'Biochimie' de Michel Guilloton et Bernadette Quintard, Paul-François Gallet, Dunod éditeur
