# Supplementary material for: Monitoring how changes in pedagogical practices have improved student interest and performance for an introductory biochemistry course
Source: FEBS Open Bio. 2018 Mar 15;8(4):494–501. doi: 10.1002/2211-5463.12409 (PMC5881536; doi:10.1002/2211-5463.12409)
Supplement: Supplementary file 4 — Data S3. Examples of MCQs, in English. [file FEB4-8-494-s004.pdf]

# Exemples of MCQ used in lectures

# Which of the following ionic forms of lysine cannot exist in aqueous solution?

1 ✓

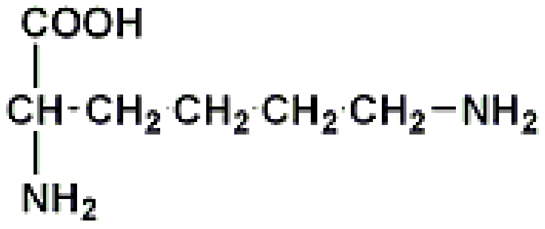

2

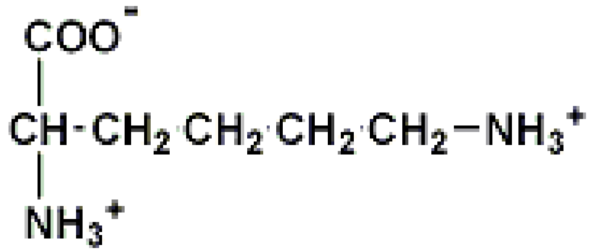

3 ✓

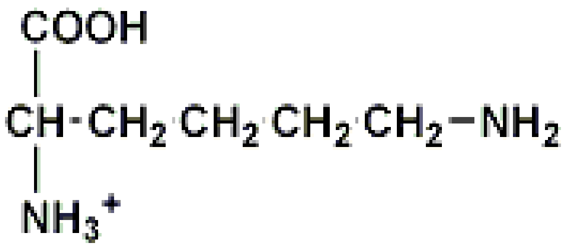

4

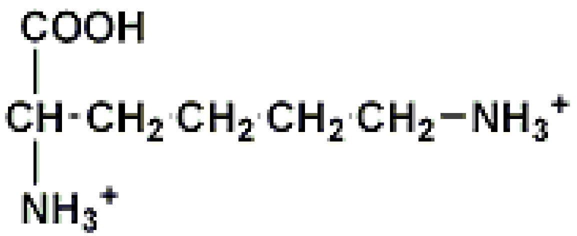

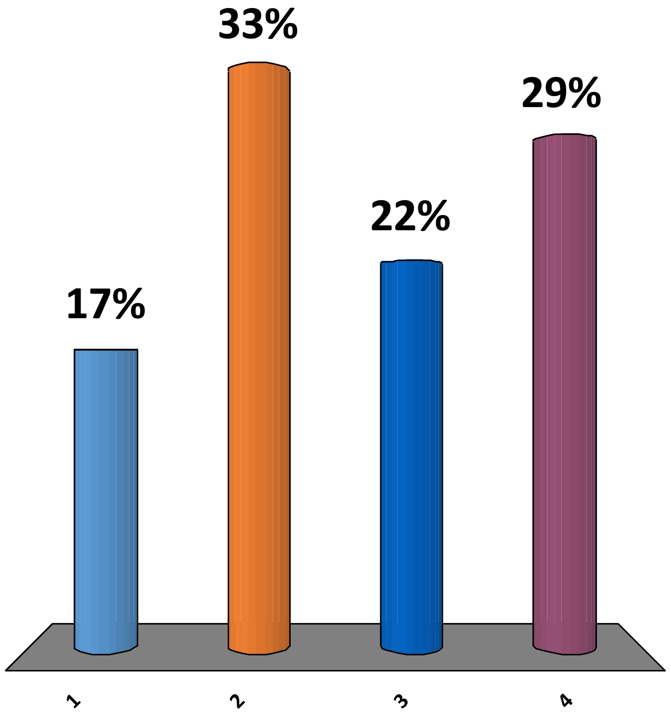


Comment :

This question allows a discussion with students about amino acid ionisation and in particular to remind that amino acids never assume the neutral form in aqueous solution

# What is the name of this amino acid?

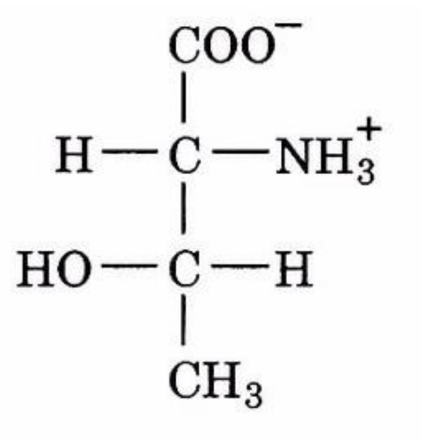

A. L-*allo*-Threonine
B. L-Threonine
✓ C. D-Threonine
D. D-*allo*-Threonine

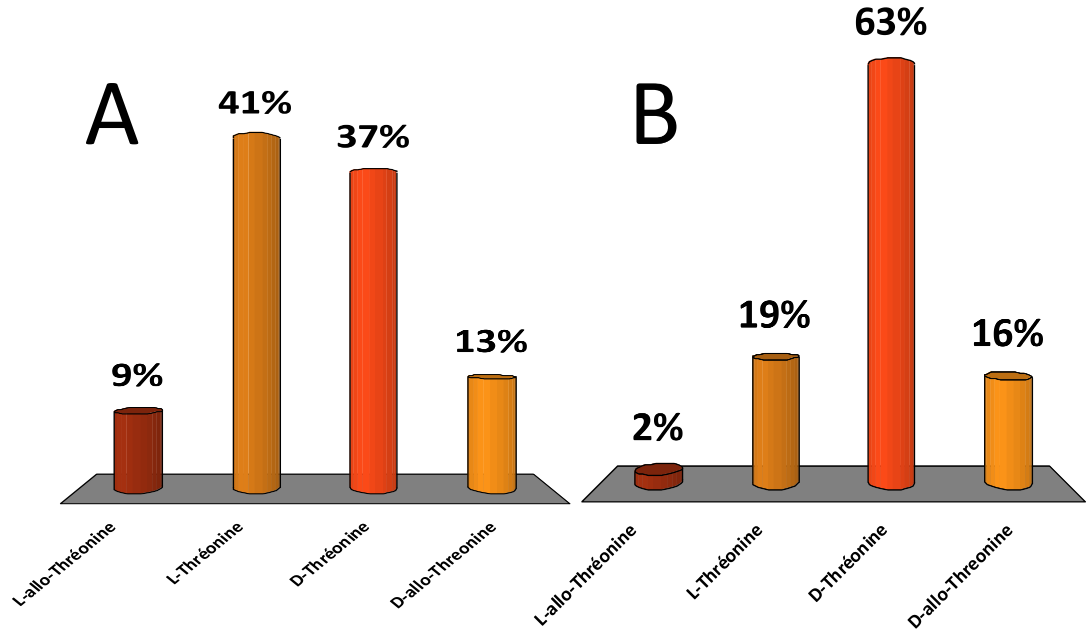


Comment :
This question was used in a peer instruction sequence: answer individually (A), then discuss with neighbouring students and answer again (B), then class-discussion.
Note the improvement between (A) and (B)

# Which of the following glycerides displays the lowest saponification index ?

1

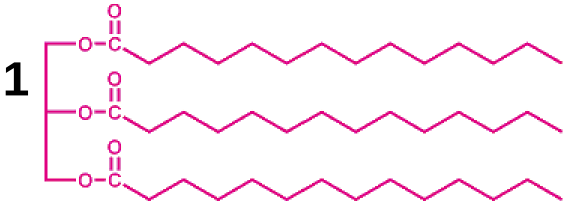

2

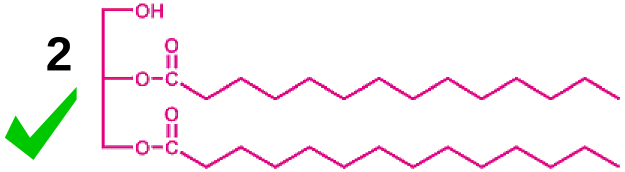


3

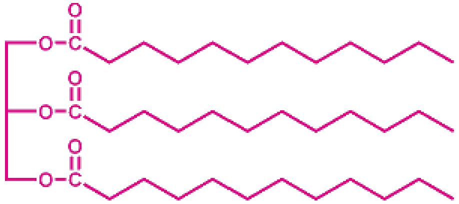

4

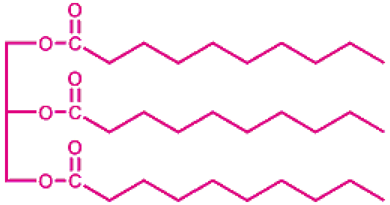

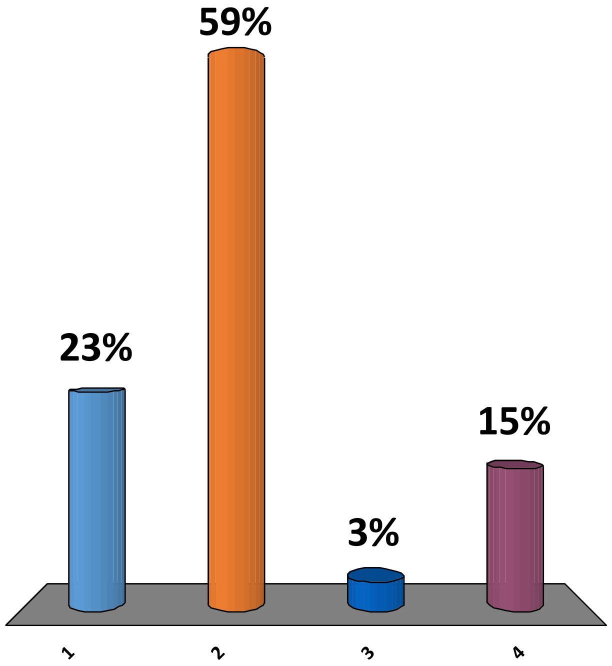


Comment :

This question was used to revise the saponification reaction and to remind that this index is useful to estimate the molecular mass of the glyceride

# Select the correct configuration for this monosacharide

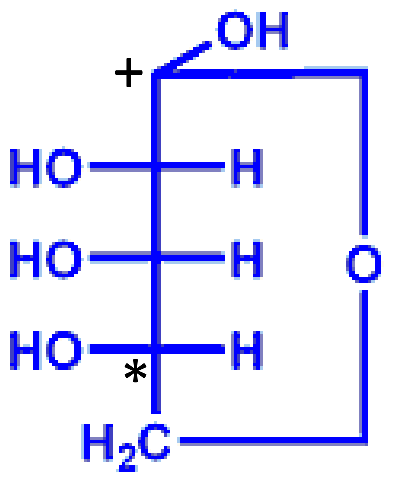


A. α-D
B. β-D
C. α-L
✓ D. β-L
E. none

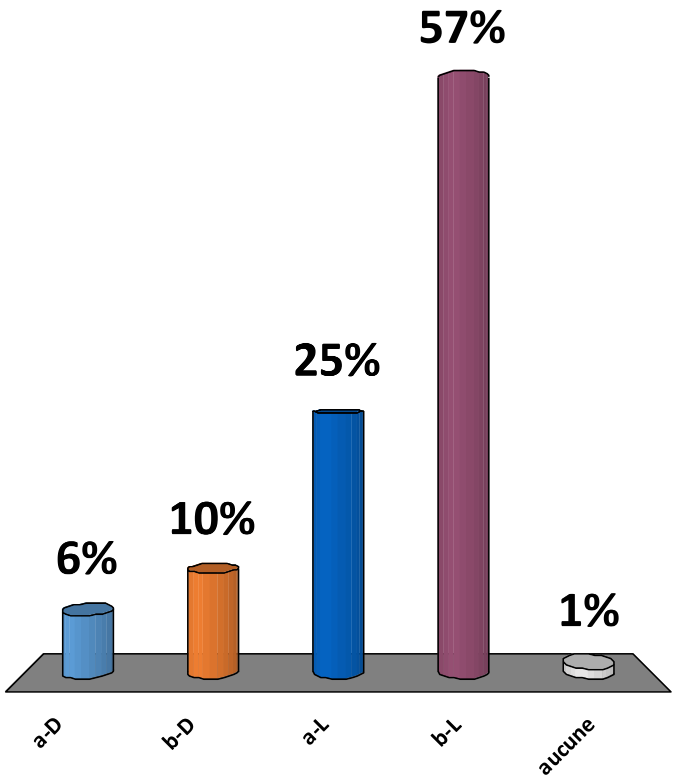


Comment :
This question allows to revise the anomeric position and the absolute configuration: the reference to determine the absolute configuration is the orientation of the hydroxyl at the asymetric center (*) farthest from its carbonyl group (+)
