# Supplementary material for: Monitoring how changes in pedagogical practices have improved student interest and performance for an introductory biochemistry course
Source: FEBS Open Bio. 2018 Mar 15;8(4):494–501. doi: 10.1002/2211-5463.12409 (PMC5881536; doi:10.1002/2211-5463.12409)
Supplement: Supplementary file 5 — Data S4. Form of the intermediate SET, in English and French. [file FEB4-8-494-s005.pdf]

Université d’Artois

Professor ___**Y KARAMANOS**_

Intermediate SET

UE ________**BBM1**______

The purpose of this evaluation is to give me elements that will allow me to improve the course and the way I teach it to you, so as to help you with your learning. I will be the only one to see your answers and I will share with you the summary of your observations

1. What are the strong aspects of this course and of the way I teach it to you? In other words, what contributes most to your learning?

2. What specific suggestions do you have for changes I can make to improve the course and of the way to teach it to you?

3. Is the rhythm of the course?

Too fast adequate too slow

Anonyme

Université d'Artois

Professeur ___**Y KARAMANOS**_

Evaluation intermédiaire du cours

UE _______**BBM1**_____

Le but de vos remarques/impressions est de me donner des éléments qui me permettront d'améliorer le cours ainsi que la manière de vous l'enseigner, de façon à vous aider pour vos apprentissages. Je serai le seul à voir vos réponses et je vous ferai part de la synthèse de vos observations

1. Quels sont les aspects forts de ce cours et de la manière de vous l'enseigner ? En d'autres termes qu'est-ce qui contribue le plus à vos apprentissages ?

2. Quelles suggestions spécifiques avez-vous pour des changements que je peux faire pour améliorer le cours et de la manière de vous l'enseigner ?

3. Est-ce que le rythme du cours est :

trop rapide juste comme il faut trop lent

Anonyme
