# Supplementary material for: Monitoring how changes in pedagogical practices have improved student interest and performance for an introductory biochemistry course
Source: FEBS Open Bio. 2018 Mar 15;8(4):494–501. doi: 10.1002/2211-5463.12409 (PMC5881536; doi:10.1002/2211-5463.12409)
Supplement: Supplementary file 6 — Data S5. Form of the final SET, in English and French. [file FEB4-8-494-s006.pdf]

Student Evaluation of Teaching (SET)

Teaching Unit **BBM1**

The purpose of this evaluation is to give me elements that will allow me to improve the course and the way I teach it to you, so as to help you with your learning. I will be the only one to see your answers and I will share with you the summary of your observations

Organisation

	Strongly agree	somewhat agree	Somewhat disagree	Strogly disagree
1. From the beginning the learning targets have been well defined	☐	☐	☐	☐
2. From the beginning, personal investment and work load has been specified	☐	☐	☐	☐
3. The planned program was done entirely	☐	☐	☐	☐
4. The lectures were well organised	☐	☐	☐	☐
5. The tutorials were well organised	☐	☐	☐	☐
6. The moodle resources were well organised	☐	☐	☐	☐

knowledge/skills taught

7. I believe this course allowed me to progress	☐	☐	☐	☐
8. I believe this course prepares me for future professional life	☐	☐	☐	☐

Assessment rules

9. I am clearly informed about the assessment rules and the grading plan	☐	☐	☐	☐
10. I prefer to be informed of my grades individually	☐	☐	☐	☐

interest on teaching/pedagogical methods

11. I understood the interest of this course for the curriculum	☐	☐	☐	☐
12. I was brought to use the content of the course to solve practical problems and interpret real situations	☐	☐	☐	☐
13. This course developed my interest for biochemistry	☐	☐	☐	☐
14. The moodle resources helped me with learning	☐	☐	☐	☐

Your profile

From the beggining I attended:

☐1 ☐ 2 ☐3 ☐4 ☐5 ☐6 ☐7 ☐8 ☐9 ☐10 ☐11 lectures

☐1 ☐ 2 ☐3 ☐4 ☐5 ☐6 ☐7 ☐8 ☐9 ☐10 ☐11 tutorials

I am enrolled at the 1st year of licence for:

☐ the first time ☐ the second time ☐ the third time or more.

What are the points of the course that you are most interested in?

Have you experienced difficulties ? Have you suggestions to improve the course?

Anonyme

Evaluation de l'unité d'enseignement

BBM1

Le but de cette évaluation est de me donner des éléments qui me permettront d'améliorer le cours ainsi que la manière de vous l'enseigner, de façon à vous aider pour vos apprentissages. Je serai le seul à voir vos réponses et je vous ferai part de la synthèse de vos observations

Organisation de l'enseignement

	complètement d'accord	plutôt d'accord	plutôt pas d'accord	Absolument pas d'accord
1. Dès le début les objectifs ont été bien définis	☐	☐	☐	☐
2. Dès le début de l'enseignement, la part d'investissement et de travail personnel a été précisée	☐	☐	☐	☐
3. Le programme annoncé a été traité entièrement	☐	☐	☐	☐
4. L'organisation des cours a été correcte	☐	☐	☐	☐
5. L'organisation des TD a été correcte	☐	☐	☐	☐
6. L'organisation des ressources sur ENT (moodle) a été correcte	☐	☐	☐	☐

Connaissances / compétences enseignées

7. J'ai le sentiment que cet enseignement m'a permis de progresser	☐	☐	☐	☐
8. J'ai le sentiment qu'il me prépare à la vie professionnelle	☐	☐	☐	☐

Contrôle des connaissances

9. Je suis clairement informé(e) des modalités de contrôle des connaisances	☐	☐	☐	☐
10. Je prefère être informé de ma note au partiel à titre individuel	☐	☐	☐	☐

Intérêt suscité par l'enseignement / méthodes pédagogiques

11. J'ai compris l'intérêt de cet enseignement pour ma formation	☐	☐	☐	☐
12. J'ai été amené(e) à utiliser le contenu de l'enseignement pour résoudre des problèmes pratiques et interpréter des situations réelles	☐	☐	☐	☐
13. L'enseignement a développé mon intérêt pour la matière enseignée	☐	☐	☐	☐
14. Les ressources moodle m'ont aidé pour mes apprentissages	☐	☐	☐	☐

Votre profil

Depuis le début de cet enseignement j'ai assisté à :
Je suis inscrit dans cette année d'étude pour :

Quels sont les points des cours/TD/moodle qui vous ont le plus intéressé ?

Avez-vous eu des difficultés ? Des suggestions pour améliorer cours/TD/moodle ?

Anonyme
